# The efficacy of kinesiology tape for rotator cuff injuries: a meta-analysis of randomized trials

**DOI:** 10.3389/fmed.2025.1695350

**Published:** 2025-11-10

**Authors:** Yuan Luo, Liyue Zhang, Zhongbao Tang, Li Chen, Dingfan Zhou, Haiyan Huang

**Affiliations:** 1 The First People's Hospital of Neijiang City, Neijiang, Sichuan, China; 2 Southwest Medical University of China, Luzhou, China; 3 Shuangcai Central Health Center, Neijiang, Sichuan, China

**Keywords:** rotator cuff injuries, kinesiology tape, systematic review, meta-analysis, pain

## Abstract

**Objective:**

Kinesiology tape has shown certain effects in treating rotator cuff injuries, but its efficacy remains controversial. This study aims to evaluate the effectiveness of kinesiology tape in treating rotator cuff injuries through a systematic review and meta-analysis.

**Methods:**

We conducted a search in PubMed, Embase, Web of Science, Cochrane Library, CNKI, Wanfang Data and VIP databases, with the search period ending in August 2025. Randomized controlled trials (RCTs) comparing KT with comparable single rehabilitation interventions, placebos or traditional rehabilitation were included. The outcomes included shoulder pain, range of motion, and upper limb movement.

**Results:**

A total of 17 studies (959 participants) were included. Kinesiology tape (KT) showed a significant therapeutic effect on the shoulder pain score (MD = −0.94; 95% CI: [−1.27, −0.95], *Z* = 5.6, *p* < 0.0001, *I*^2^ = 75%). KT significantly increased the shoulder flexion range of motion (MD = 9.24; 95% CI: [3.11, 15.36], *Z* = 2.96, *p* < =0.003, *I*^2^ = 91%). KT significantly increased the shoulder abduction range of motion (MD = 9.14; 95% CI: [6.99, 11.29], *Z* = 8.34, *p* < 0.0001, *I*^2^ = 38%). KT significantly improved upper limb function (MD = –4.38; 95% CI: [−5.19, −3.57], *Z* = 10.64, *p* < 0.0001, *I*^2^ = 19%). Through subgroup analysis based on pain assessment, the differences in therapeutic effects of KT treatment were further explored in different types of control groups, treatment cycles, stimulation areas, and genders.

**Conclusion:**

The results of this meta-analysis indicate that KT has a significant positive effect in alleviating shoulder pain and upper limb dysfunction in patients with rotator cuff injuries. However, due to the presence of potential risk factors, the therapeutic effect of KT needs to be interpreted with caution.

## 1 Introduction

Rotator cuff injuries (RCIs) represent a prevalent musculoskeletal disorder affecting approximately 20–30% of the general adult population, with incidence markedly increasing with age (1, 2). These injuries encompass a spectrum of pathologies ranging from tendinopathy and partial-thickness tears to full-thickness tears, frequently resulting in persistent shoulder pain, functional impairment, diminished range of motion (ROM), and significant reductions in quality of life and occupational capacity (3–5). Conventional therapeutic approaches include physical therapy, non-steroidal anti-inflammatory drugs (NSAIDs), corticosteroid injections, and surgical intervention (6, 7). However, limitations such as variable efficacy, potential adverse effects of pharmacotherapy, procedural risks associated with injections and surgery, and substantial healthcare costs underscore the ongoing need for effective, accessible, and low-risk adjunctive or alternative treatments.

Kinesiology tape (KT), an elastic therapeutic tape engineered to mimic human skin’s elasticity, has gained widespread empirical and clinical traction over the past two decades, particularly within sports medicine and rehabilitation settings (8, 9). Supporters believe that KT exerts its therapeutic effects through various biomechanical and neurophysiological mechanisms. It can provide proprioceptive feedback to enhance neuromuscular control and the position of the scapula (10, 11). By pulling the skin, it promotes microcirculation and lymphatic drainage (12). Additionally, it regulates pain perception through gating theory, providing subtle support for fatigued or injured muscles while not restricting joint movement (13). Therefore, KT is widely used in the treatment of peripheral nerve injury, with its main goal being to relieve pain, restore functional activity ability, and improve activities of daily living (14, 15).

Although KT has been widely used in clinical practice, its therapeutic effect on rotator cuff injuries remains controversial. Although numerous randomized controlled trials have investigated the impact of KT on pain intensity, shoulder function outcomes, arm, shoulder and hand disability questionnaires or the objective measurements of shoulder pain and dysfunction index, as well as joint range of motion, the results vary (16–18). Some trials report that the group treated with KT shows statistically significant and clinically meaningful improvements, whether as a standalone intervention or as an adjunct to conventional physical therapy (19, 20). In contrast, other well-designed randomized controlled trials indicate that compared with sham ligation, placebo intervention or standard care alone, kinesiology tape has little or no significant benefits (21). This inconsistency may stem from methodological differences in different studies, including ligation techniques, participant characteristics, control intervention measures, outcome measurement standards, and follow-up time.

Although previous systematic reviews and meta-analyses have attempted to synthesize the evidence on KT for shoulder pathologies, significant knowledge gaps persist. Earlier syntheses often included studies with mixed shoulder conditions (e.g., adhesive capsulitis, impingement syndrome alongside RCIs), potentially diluting injury-specific effects (22–25). Others incorporated non-randomized or lower-quality studies, introducing potential bias (26). Critically, several new high-quality RCTs focusing specifically on RCIs have been published in recent years, necessitating an updated and more focused quantitative synthesis. Furthermore, the existing meta-analyses often overlook crucial subgroup analyses, analyses of the effects of different stimulation areas or the duration of fixation, which are of vital importance for guiding evidence-based clinical practice.

## 2 Materials and methods

This meta-analysis adhered to the Preferred Reporting Items for Systematic Reviews and Meta-Analyses guidelines. Ethical approval was not requisite, given that this study constitutes secondary research based on previously published articles. No conflicts of interest were present among the authors.

### 2.1 Literature search strategy

Two investigators conducted an electronic literature search independently to evaluate the results of the KT treatment for rotator cuff injuries. As of August 2025, the electronic databases included in the search were PubMed, Embase, the Web of Science, the Cochrane Library, the Wanfang Database, the China Journal Full-Text Database (CNKI), and the VIP Database, with the search terms limited to English and Chinese. The following search terms were used: “Kinesio Tape,” “Athletic Tape,” “Tape, Athletic,” “kinesiology tape,” “Orthotic Tape,” “Rotator Cuff Injuries,” “Cuff Injury, Rotator,” “Injury, Rotator Cuff,” “Rotator Cuff Injury,” “Rotator Cuff Tears,” “Tear, Rotator Cuff,” and so forth. Detailed search results can be found in Table 1.

**Table 1:** PubMed: session results.

Number	Query	Search details	Results
#7	#3 AND #6	((“Rotator Cuff Injuries”[Mesh]) OR ((((((((((((((((((((Rotator Cuff Injuries) OR (Cuff Injury, Rotator)) OR (Injury, Rotator Cuff)) OR (Rotator Cuff Injury)) OR (Rotator Cuff Tears)) OR (Rotator Cuff Tear)) OR (Tear, Rotator Cuff)) OR (Tears, Rotator Cuff)) OR (Rotator Cuff Tendinitis)) OR (Rotator Cuff Tendinitides)) OR (Tendinitis, Rotator Cuff)) OR (Rotator Cuff Tendinosis)) OR (Rotator Cuff Tendinoses)) OR (Tendinoses, Rotator Cuff)) OR (Tendinosis, Rotator Cuff)) OR (Glenoid Labral Tears)) OR (Glenoid Labral Tear)) OR (Labral Tear, Glenoid)) OR (Labral Tears, Glenoid)) OR (Tear, Glenoid Labral))) AND ((“Athletic Tape”[Mesh]) OR (((((((((Athletic Tape) OR (Tape, Athletic)) OR (Orthotic Tape)) OR (Tape, Orthotic)) OR (Kinesio Tape)) OR (Kinesio Tapes)) OR (Tape, Kinesio)) OR (Tapes, Kinesio)) OR (Kinesiotape)))	18
#6	#4 OR #5	(“Rotator Cuff Injuries”[Mesh]) OR ((((((((((((((((((((Rotator Cuff Injuries) OR (Cuff Injury, Rotator)) OR (Injury, Rotator Cuff)) OR (Rotator Cuff Injury)) OR (Rotator Cuff Tears)) OR (Rotator Cuff Tear)) OR (Tear, Rotator Cuff)) OR (Tears, Rotator Cuff)) OR (Rotator Cuff Tendinitis)) OR (Rotator Cuff Tendinitides)) OR (Tendinitis, Rotator Cuff)) OR (Rotator Cuff Tendinosis)) OR (Rotator Cuff Tendinoses)) OR (Tendinoses, Rotator Cuff)) OR (Tendinosis, Rotator Cuff)) OR (Glenoid Labral Tears)) OR (Glenoid Labral Tear)) OR (Labral Tear, Glenoid)) OR (Labral Tears, Glenoid)) OR (Tear, Glenoid Labral))	15,856
#5	“rotator cuff injuries”[MeSH Terms] OR (“rotator”[All Fields] AND “cuff”[All Fields] AND “injuries”[All Fields]) OR “rotator cuff injuries”[All Fields] OR (“tear”[All Fields] AND “glenoid”[All Fields] AND “labral”[All Fields]) OR “tear, glenoid labral”[All Fields]	(((((((((((((((((((Rotator Cuff Injuries) OR (Cuff Injury, Rotator)) OR (Injury, Rotator Cuff)) OR (Rotator Cuff Injury)) OR (Rotator Cuff Tears)) OR (Rotator Cuff Tear)) OR (Tear, Rotator Cuff)) OR (Tears, Rotator Cuff)) OR (Rotator Cuff Tendinitis)) OR (Rotator Cuff Tendinitides)) OR (Tendinitis, Rotator Cuff)) OR (Rotator Cuff Tendinosis)) OR (Rotator Cuff Tendinoses)) OR (Tendinoses, Rotator Cuff)) OR (Tendinosis, Rotator Cuff)) OR (Glenoid Labral Tears)) OR (Glenoid Labral Tear)) OR (Labral Tear, Glenoid)) OR (Labral Tears, Glenoid)) OR (Tear, Glenoid Labral)	15,856
#4	Rotator Cuff Injuries”[MeSH Terms]	“Rotator Cuff Injuries”[Mesh] Sort by: Most Recent	8,865
#3	#1 OR #2	(“Athletic Tape”[Mesh]) OR (((((((((Athletic Tape) OR (Tape, Athletic)) OR (Orthotic Tape)) OR (Tape, Orthotic)) OR (Kinesio Tape)) OR (Kinesio Tapes)) OR (Tape, Kinesio)) OR (Tapes, Kinesio)) OR (Kinesiotape))	1,538
#2	athletic tape[MeSH Terms] OR (“athletic”[All Fields] AND “tape”[All Fields]) OR “athletic tape”[All Fields] OR “kinesiotape”[All Fields] OR “kinesiotaping”[All Fields]	((((((((Athletic Tape) OR (Tape, Athletic)) OR (Orthotic Tape)) OR (Tape, Orthotic)) OR (Kinesio Tape)) OR (Kinesio Tapes)) OR (Tape, Kinesio)) OR (Tapes, Kinesio)) OR (Kinesiotape)	1,538
#1	“Athletic Tape”[MeSH Terms]	Search: “Athletic Tape”[Mesh] Sort by: Most Recent	997

### 2.2 Inclusion and exclusion criteria

Inclusion criteria: (1) Research design: randomized controlled trial; (2) Patient population: rotator cuff injury; (3) Intervention measures: exercise therapy; (4) Control group: placebo, sham therapy or conventional rehabilitation; (5) Outcomes: pain, joint range of motion and function.

Exclusion criteria: (1) Exclude cases from animal experiments; (2) Exclude cases with incomplete data; (3) Exclude cases that received improper intervention measures; (4) Exclude cases from non-randomized controlled trials.

### 2.3 Literature review and data extraction

The data were independently extracted by two reviewers, while the third reviewer integrated these data. A data extraction form had been pre-designed. The valid extracted data included: (1) research data, such as authors, methods, years and countries; (2) population characteristics, including sample size, gender and age; (3) intervention methods, stimulus range; (4) outcome indicators, extracting average values and standard deviations of pain scores, upper limb function scores, shoulder flexion and abduction range of motion.

### 2.4 Evaluation of the quality of the literature

Risk of bias assessment across the included studies was performed using the Cochrane Risk of Bias tool. This evaluation systematically appraised potential sources of bias in seven critical domains: random sequence generation, allocation concealment, blinding of participants and personnel, blinding of outcome assessment, incomplete outcome data, selective reporting, and other potential sources of bias.

### 2.5 Statistical analyses

Statistical analyses were conducted utilizing RevMan 5.3 (The Cochrane Collaboration) and Stata 17.0 (StataCorp LP). Study heterogeneity was evaluated primarily via the Chi-square test. A fixed-effect model was employed for analyses demonstrating low heterogeneity (*I*^2^ ≤ 50%). In instances of substantial heterogeneity (*I*^2^ > 50%), a random-effects model was implemented. Subsequent investigations into heterogeneity sources were performed through subgroup analyses, sensitivity analyses, and meta-regression. Continuous outcome variables are expressed as mean differences (MD) with corresponding 95% confidence intervals (CI). Statistical significance was established at a two-sided *p*-value threshold of < 0.05 (27).

## 3 Results

### 3.1 Results of the literature search

**Figure 1:**
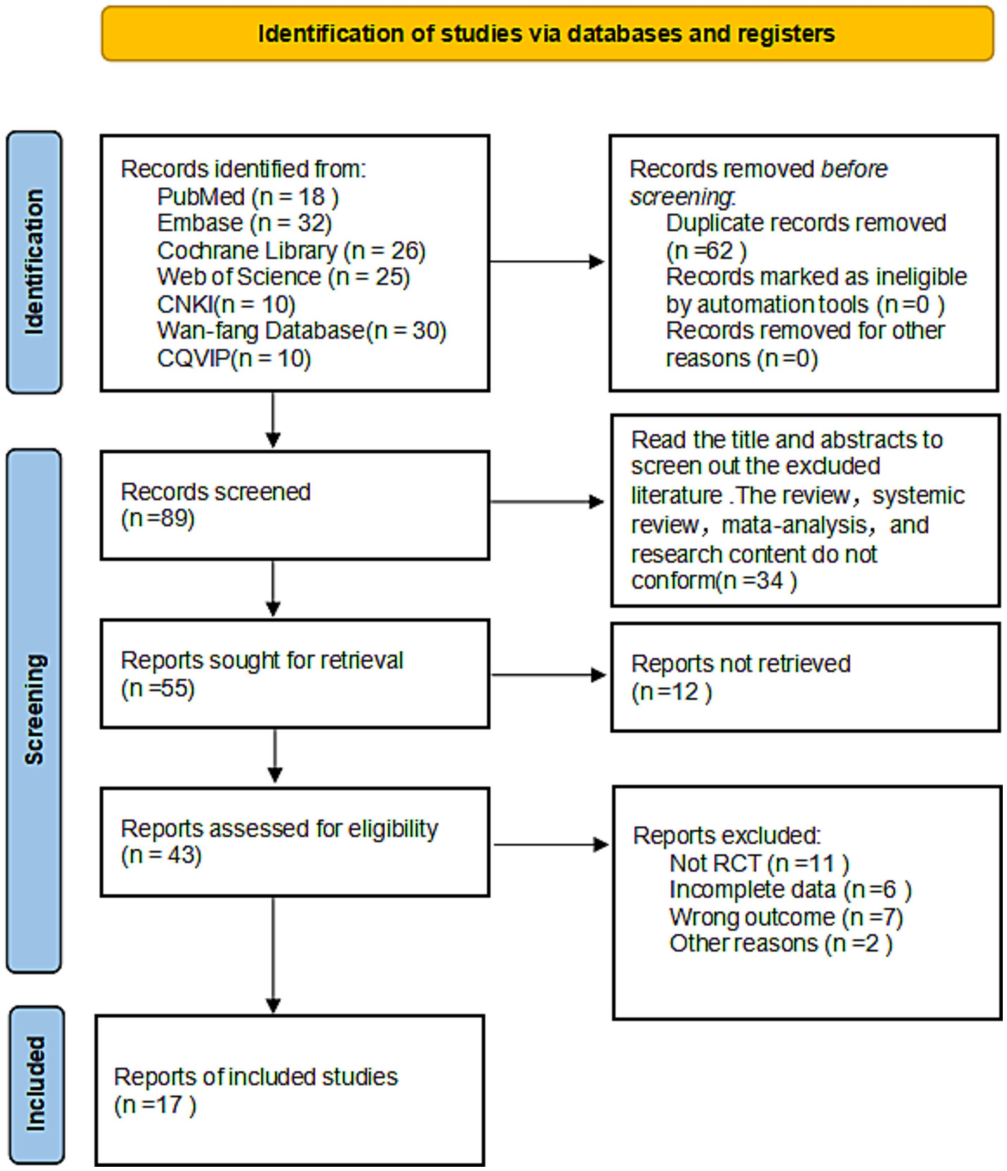
Flowchart of literature screening.

The literature search and screening process is depicted in Figure 1. Initial searches across relevant databases yielded 151 potentially eligible publications (101 English-language articles: PubMed *n* = 18, Embase *n* = 32, Web of Science *n* = 25, Cochrane Library *n* = 26; 50 Chinese-language articles: CNKI *n* = 10, Wanfang *n* = 30, VIP *n* = 10). Following import into EndNote software, 62 duplicate records were identified and removed. Subsequent screening of titles and abstracts resulted in the exclusion of a further 72 articles. Ultimately, 17 randomized controlled trials (RCTs) investigating the use of KT for rotator cuff injuries met the inclusion criteria (28–44). No additional relevant articles were identified from other sources. Two researchers independently performed the screening process, with consensus achieved at all stages.

### 3.2 Basic characteristics and quality assessment of included studies

**Figure 2:**
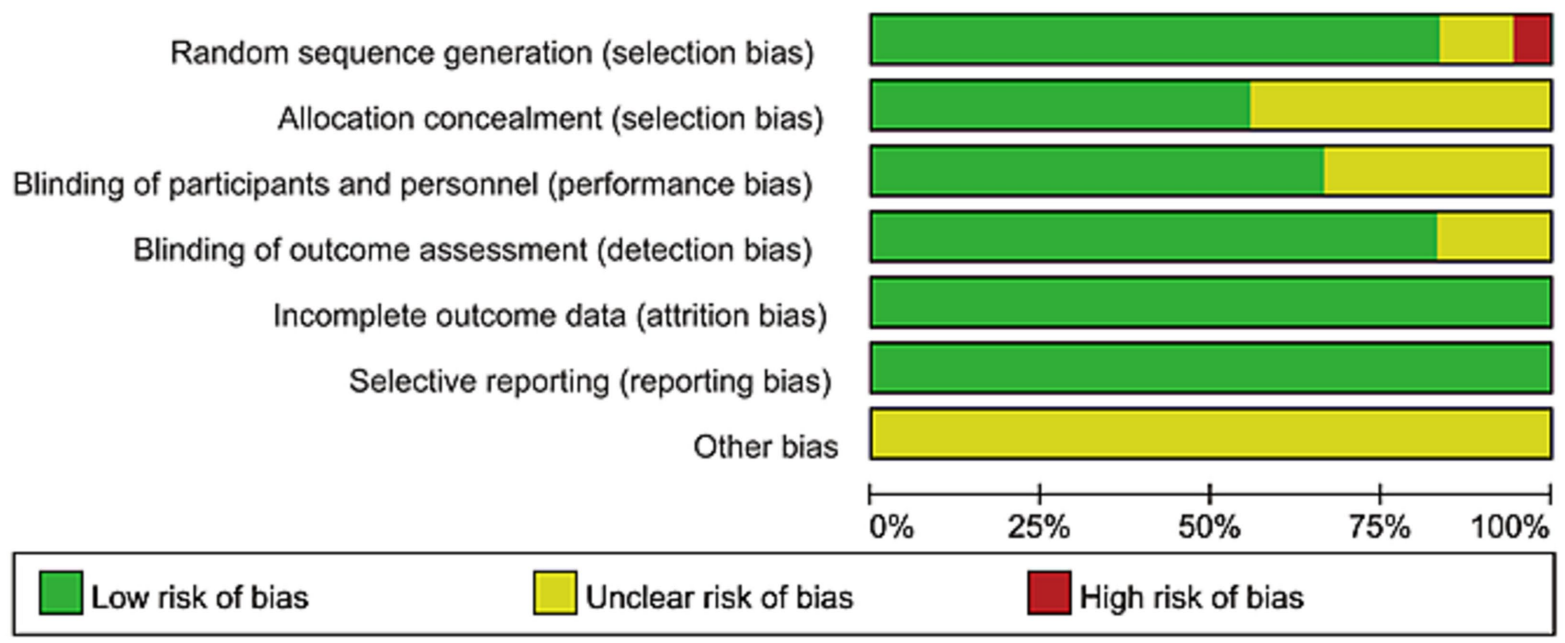
Risk of bias graph.

**Figure 3:**
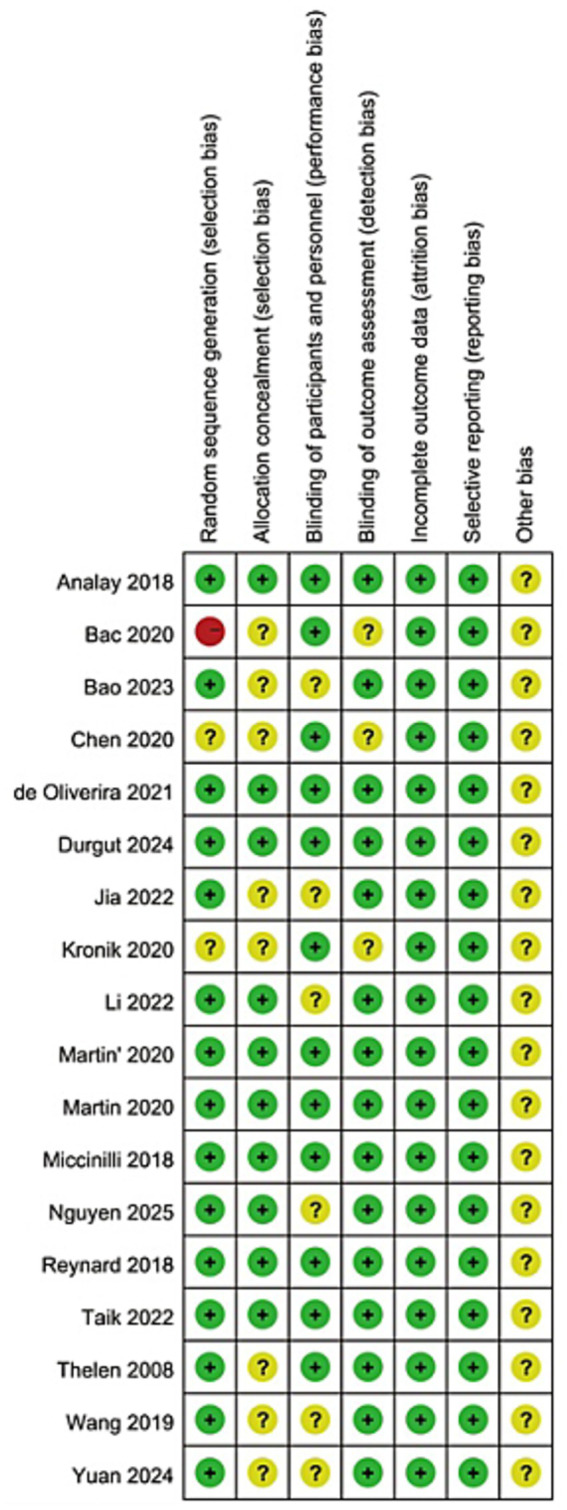
Risk of bias summary.

The characteristics of the included studies are summarized in Table 2. This systematic review incorporated a total of 17 randomized controlled trials (RCTs), comprising 11 (28–38) English-language and 6 (39–44) Chinese-language publications. Data from 959 patients diagnosed with rotator cuff injuries were analyzed (436 males, 523 females). Sample sizes across the studies ranged from 39 to 92 participants. Regarding interventions, KT was applied as the sole intervention in the experimental groups of 6 (28, 31, 32, 36–38) RCTs, while the remaining RCTs combined KT with other physical therapies. For outcome measures, pain was assessed in 15 (28–32, 34, 36–44) studies, shoulder flexion range of motion (FLE) in 12 (29, 31, 34–41, 43, 44) studies, shoulder abduction range of motion (ABD) in 11 (29, 34–41, 43, 44) studies, and the Disabilities of the Arm, Shoulder and Hand (DASH) questionnaire was reported in 6 (29, 30, 32, 33, 36, 38) studies. The risk of bias assessment for the included RCTs was conducted according to the Cochrane Handbook for Systematic Reviews of Interventions, evaluating seven specific domains. In the assessment of the risk of bias for 17 studies, 1 (33) study showed a relatively high risk of bias in at least one aspect, while the other 16 (28–32, 34–44) studies indicated that the risk of bias was unclear in at least one aspect. Overall, the risk of bias rating for these studies was “unclear,” due to the insufficiently detailed reporting of the concealment of allocation. Most studies did not mention the relevant details of blinding, resulting in the classification of most studies’ blinding as “unclear.” As shown in Figures 2, 3.

**Table 2:** The general date of the included studies.

Inclusion study	Yr	Intervention measure	Design	Age	Gender (female/male)	*N*	Stimulation area	Period	Outcome	Country
De Oliveira (20)	2021	KT + RET/RET	2-arm RCT	30.9 ± 9 29.4 ± 7.5	11/15 11/15	26/26	Trapezius, supraspinatus, deltoid muscle	6WK	VAS, DASH, FLE, ABD	Canada
Reynard (31)	2018	KT/PB	2-arm RCT	59 ± 9 60 ± 10	4/16 5/14	20/19	Trapezius, supraspinatus, deltoid muscle	6WK	VAS, FLE	Switzerland
Taik (32)	2022	KT/PB	2-arm RCT	57.2 ± 6.62 57.12 ± 8.88	23/2 23/2	25/25	Supraspinatus, deltoid muscle	2WK	VAS, DASH	Morocco
Bac (33)	2020	KT + RET/RET	2-arm RCT	45.67 ± 9.29 48.17 ± 10.35	15/15 17/13	30/30	Deltoid muscle	6WK	DASH	Czech
Durgut (36)	2024	KT/CT	2-arm RCT	44 ± 5.64 44.47 ± 7.16	14/9 10/13	23/23	Trapezius, supraspinatus, deltoid muscle	1WK	NRS, DASH, FLE, ABD	Turkey
Thelen (37)	2008	KT/PB	2-arm RCT	21.3 ± 1.7 19.8 ± 1.5	2/19 4/17	21/21	Trapezius, supraspinatus, deltoid muscle	1WK	VAS, FLE, ABD	America
Miccinilli (28)	2018	KT/PB	2-arm RCT	61 ± 12 64 ± 10	12/9 10/9	21/19	Deltoid muscle	1WK	NRS	Italy
Martin (35)	2020	KT + RET/KT/RET	3-arm RCT	46.95 ± 10.74 48.65 ± 10.27 49.2 ± 13.13	12/8 14/6 16/4	20/20/20	Trapezius, supraspinatus, deltoid muscle	2WK	FLE, ABD	Brazil
Analay (38)	2018	KT/PB	2-arm RCT	48.86 ± 10.03 54.15 ± 10.22	17/12 18/9	29/27	Deltoid muscle	1WK	VAS, DASH, FLE, ABD	Turkey
ÇiFtçi (30)	2020	KT + RET/RET	2-arm RCT	59.72 ± 2.94 58.52 ± 5.9	39/7 25/21	46/46	Trapezius, supraspinatus, deltoid muscle	3WK	VAS, DASH	Turkey
Nguyen (34)	2025	KT + RET/RET	2-arm RCT	51.27 ± 12.64 57.6 ± 13.4	30/10 29/11	40/40	Deltoid muscle	2WK	VAS, FLE, ABD	Vietnam
Chen (39)	2020	KT + RET/RET	2-arm RCT	43.28 ± 9.27 46.35 ± 10.85	12/19 14/17	31/31	Biceps, deltoid muscles	4WK	VAS, FLE, ABD	China
Bao (40)	2023	KT + RET/RET	2-arm RCT	49.63 ± 7.6 50.17 ± 8.23	10/20 6/24	30/30	Trapezius, deltoid muscles	4WK	VAS, FLE, ABD	China
Yuan (41)	2024	KT + RET/RET	2-arm RCT	50.6 ± 8.43 53.53 ± 7.45	17/13 19/11	30/30	Biceps brachii, supraspinatus muscle, deltoid muscle	1WK	VAS, FLE, ABD	China
Wang (42)	2019	KT + RET/RET	2-arm RCT	57.1 ± 4.35 56.65 ± 4.02	12/8 14/6	20/20	Trapezius, supraspinatus, deltoid muscle	2WK	VAS	China
Jia (43)	2022	KT + RET/RET	2-arm RCT	53.7 ± 11.14 50 ± 12.63	10/10 10/10	20/20	Trapezius, supraspinatus, deltoid muscle	4WK	VAS, FLE, ABD	China
Li (44)	2022	KT + RET/RET	2-arm RCT	49.63 ± 6.68 51.27 ± 5.54	18/22 20/20	40/40	Trapezius, supraspinatus, deltoid muscle	4WK	VAS, FLE, ABD	China

DASH = Disabilities of the Arm, Shoulder and Hand questionnaire; CT = cold therapy; ESWT = Extracorporeal shock wave therapy; PB = Placebo; RET = Rehabilitation treatment; FLE = Flexion; ABD = Abduction; RCT = Randomized Controlled Trial; VAS = Visual Analogue Scale; NRS = Numerical Rating Scale.

### 3.3 Outcome

#### 3.3.1 Pain assessment

**Figure 4:**
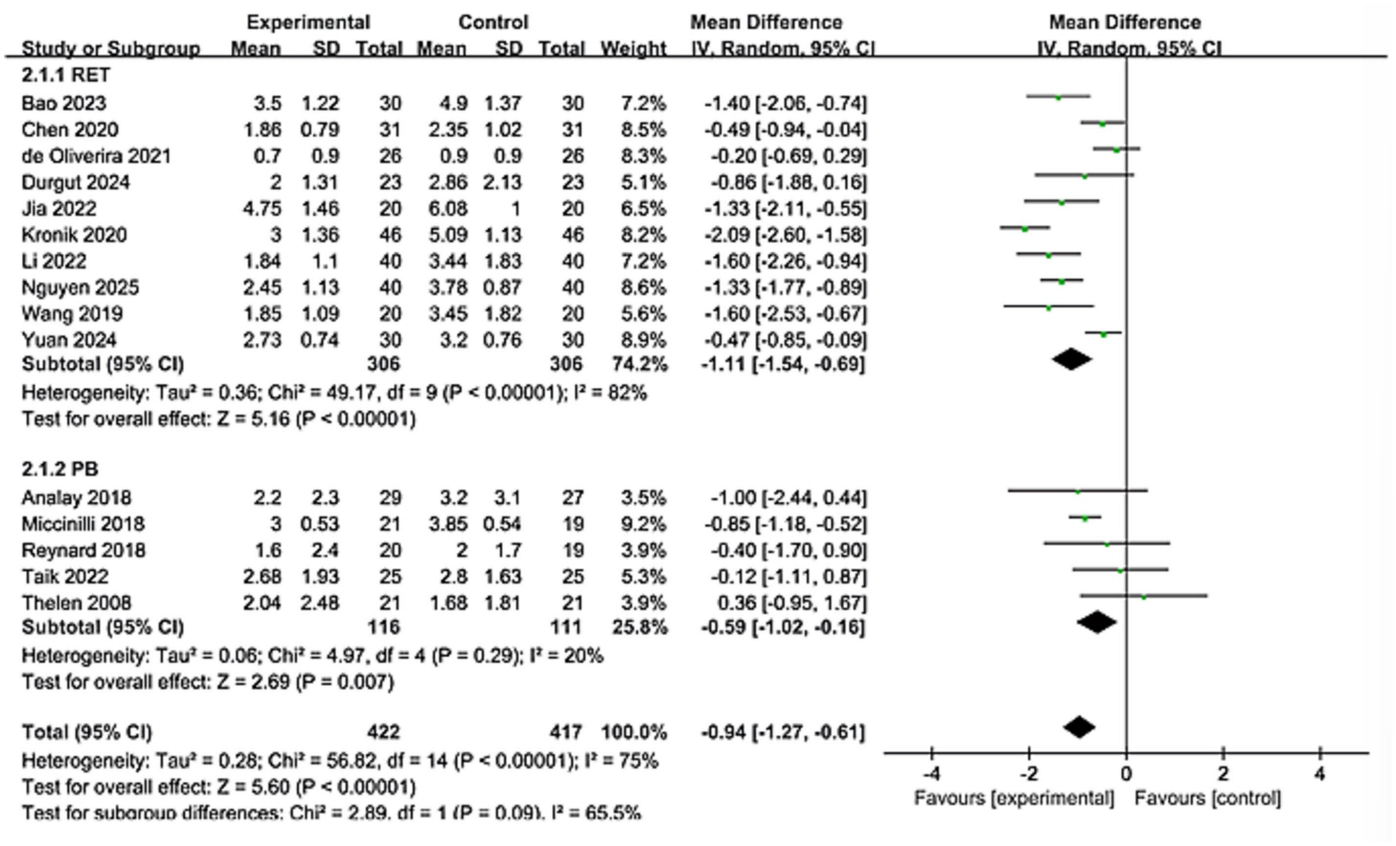
Forest plot for effectiveness of kinesio taping on changes in pain.

Pain outcomes were assessed using the Visual Analogue Scale (VAS) or the Numerical Rating Scale (NRS). The meta-analysis included 17 studies involving 839 patients. Significant heterogeneity was observed among the included studies (*I*^2^ = 75%). Consequently, a random-effects model was employed for the analysis. The results demonstrated a significantly greater reduction in pain in the KT group compared to the control group (MD = −0.94; 95% CI: [−1.27, −0.61], *Z* = 5.6, *p* < 0.0001 Figure 4). To explore potential sources of heterogeneity, subgroup analyses were performed based on intervention type, intervention duration, participant gender, and anatomical site of application (Table 3). Significant pain relief was observed in all subgroups. However, no statistically significant subgroup effects were identified for any of these variables.

**Table 3:** Effects of KT on pain scale improvement: subgroup analysis.

Variables	Group (*n*)	Participant (*n*)	MD [95%Cl]	P	I^2^%	Sub-group difference	
						*x* ^2^	*p*
Intervention measure							
PB	5	227	−0.59[−1.02,-0.16]	0.007	20%	2.89	0.09
RET	10	612	−1.11[−1.54,-0.69]	<0.00001	82%		
Period							
≥4WK	6	333	−0.9[−1.42,-0.39]	0.0006	73%	0.03	0.86
<4WK	9	506	−0.96[−1.42,-0.51]	<0.00001	78%		
Gender							
Female > Male	8	464	−1.07[−1.52,-0.61]	<0.00001	79%	0.97	0.32
Female < Male	6	799	−0.7[−1.26,-0.15]	0.010	74%		
Apply stimulation to the designated area							
Through the action of large muscle groups	9	491	−1.09[−1.64,-0.54]	<0.00001	79%	1.17	0.28
Not achieved through the action of large muscle groups	6	348	−0.74[−1.07,-0.41]	<0.00001	58%		

MD: mean difference; CI: confidence interval.

#### 3.3.2 Evaluation of shoulder flexion

**Figure 5:**
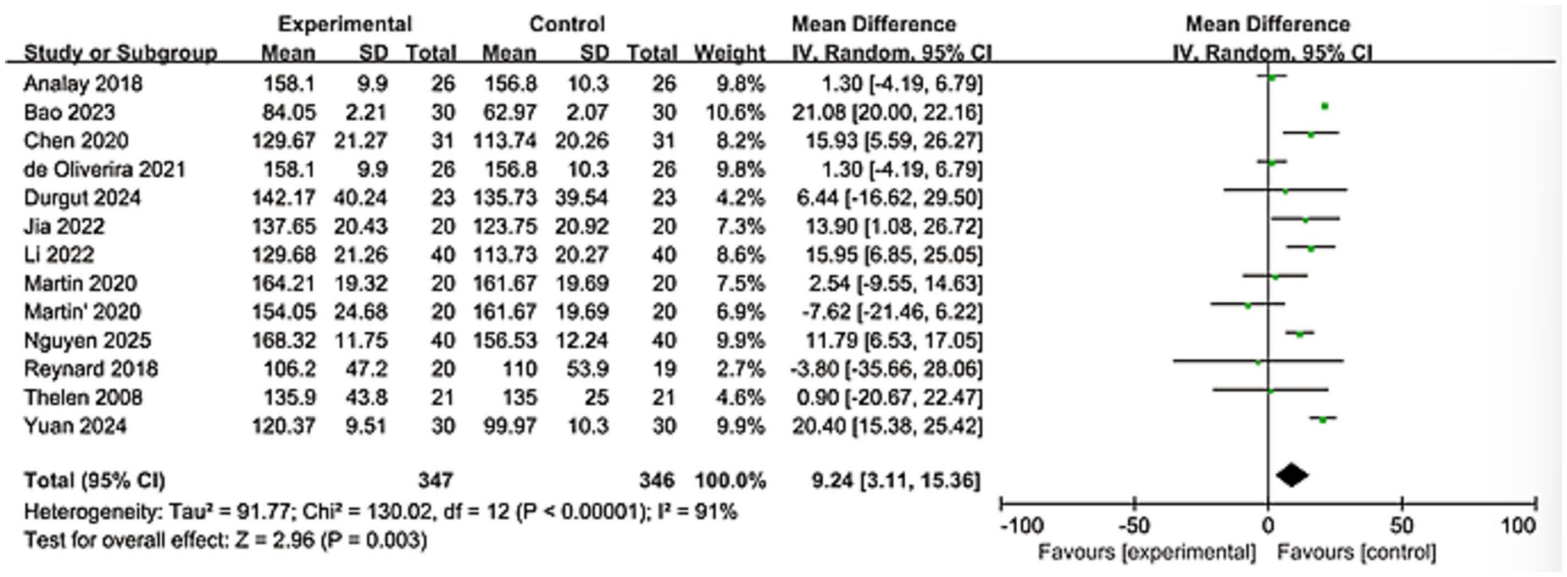
Forest plot of the effectiveness of kinesio tape on forward flexion changes.

A total of 12 studies evaluated the shoulder flexion condition, involving 693 patients. Heterogeneity analysis revealed significant differences among the studies (I^2^ = 91%). After analysis using the random effects model, the results indicated that the KT group showed better improvement in shoulder flexion compared to the control group, and the results were statistically significant (MD = 9.24; 95% CI: [3.11, 15.36], *Z* = 2.96, *p* = 0.003). As shown in Figure 5.

#### 3.3.3 Evaluation of shoulder abduction

**Figure 6:**
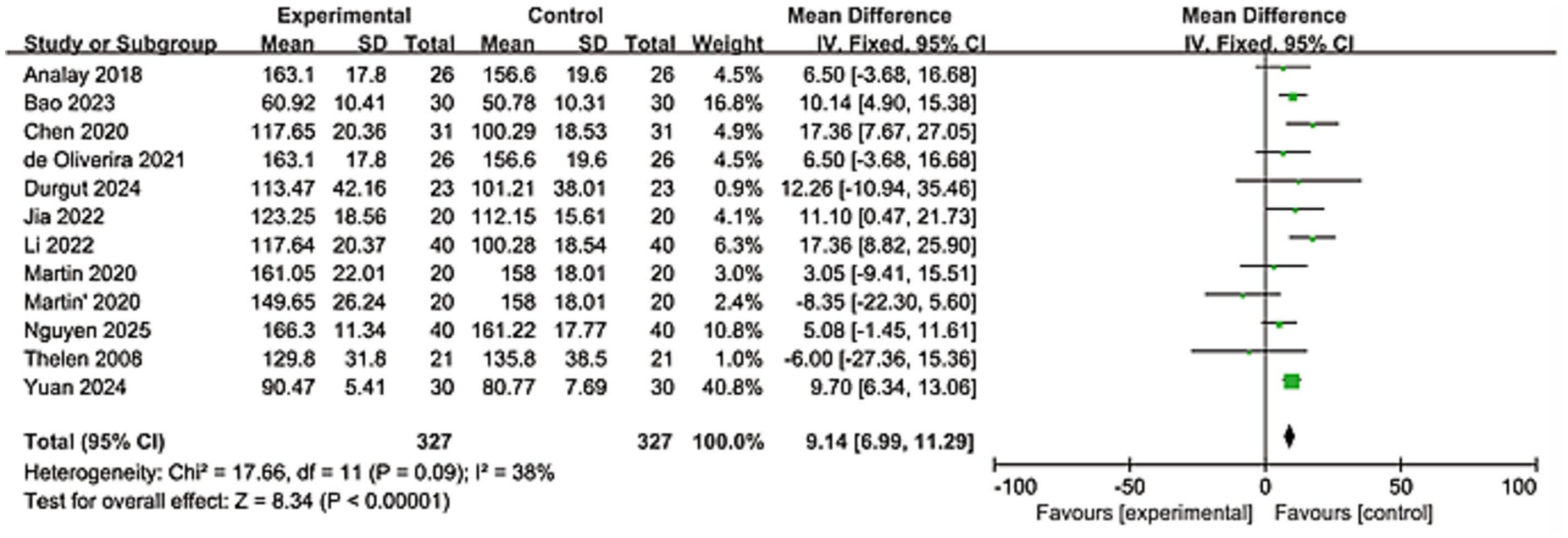
Forest plot showing the effectiveness of the kinesio taping on the external rotation change.

A total of 11 studies evaluated the shoulder abduction situation, involving 654 patients. Heterogeneity analysis showed significant differences among the studies (*I*^2^ = 38%). After analysis using the fixed-effect model, the results indicated that the KT group had better improvement in shoulder abduction compared to the control group, and the results were statistically significant (MD = 9.14; 95% CI: [6.99, 11.29], *Z* = 8.34, *p* < 0.0001). As shown in Figure 6.

#### 3.3.4 Upper limb function assessment

**Figure 7:**
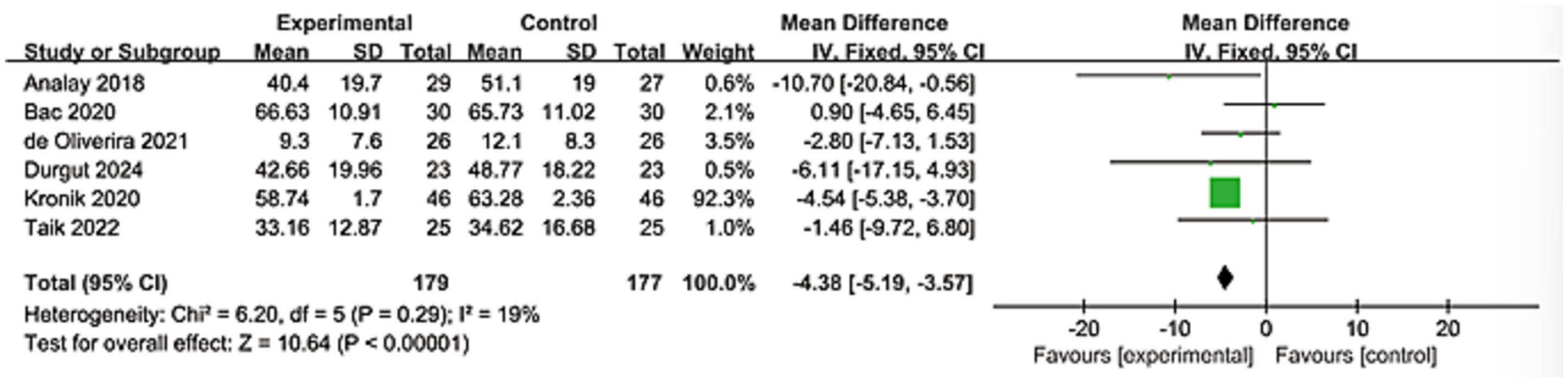
Forest plot showing the effectiveness of the kinesio taping on the changes in shoulder disability.

A total of 6 studies were included in the analysis, involving a total of 356 patients. The evaluation index was the Disabilities of the Arm, Shoulder and Hand score (DASH). Heterogeneity analysis showed significant differences among the studies (*I*^2^ = 19%). A fixed-effect model was used for analysis, and the results showed that the improvement in upper limb motor function in the KT group was better than that in the control group, and the results were statistically significant (MD = −4.38; 95% CI: [−5.19, −3.57], *Z* = 10.64, *p* < 0.0001). As shown in Figure 7.

### 3.4 Sensitivity analysis

**Figure 8:**
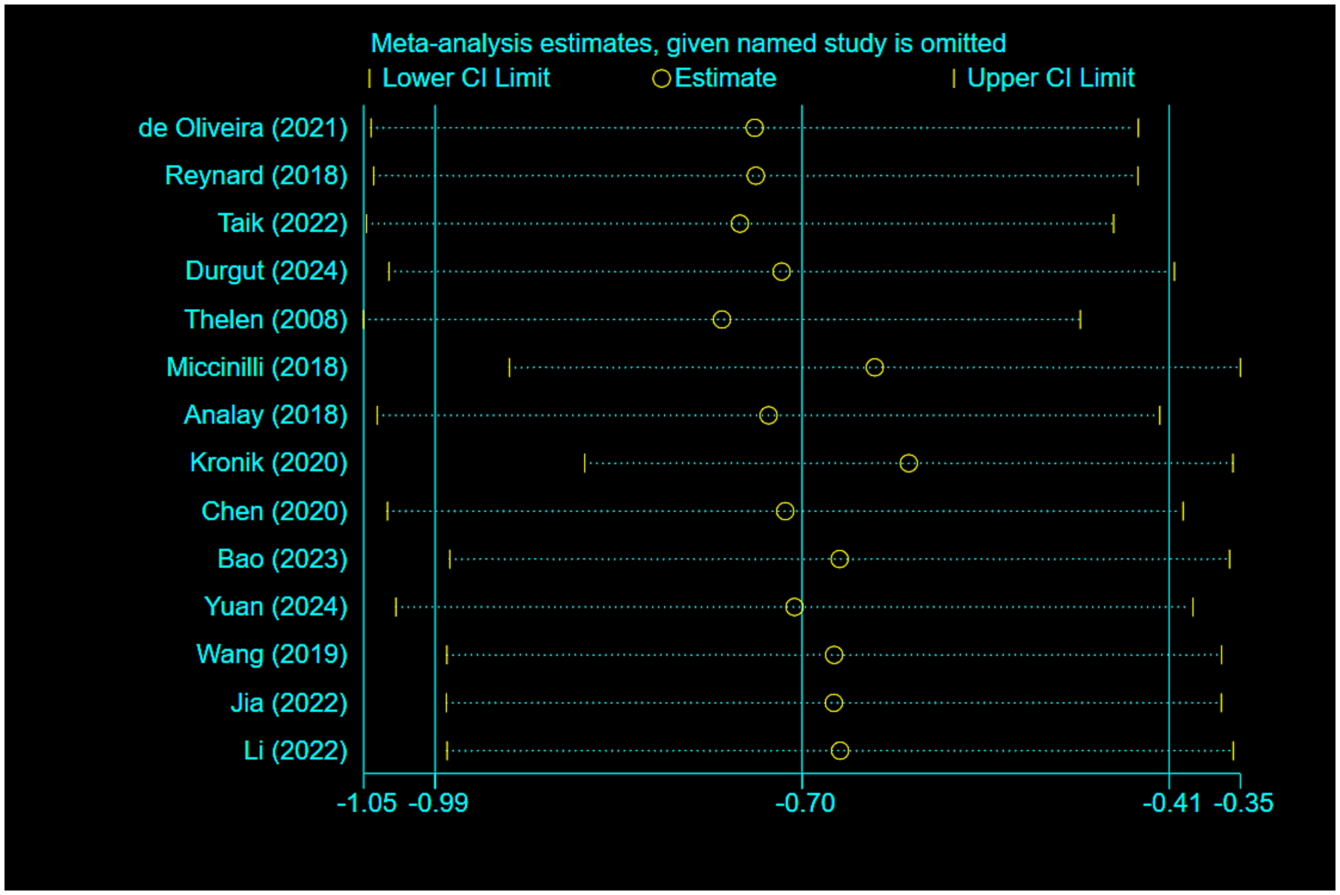
Sensitivity analysis of pain conditions in patients with rotator cuff injuries in the two groups.

Sensitivity analysis employing the leave-one-out method confirmed the stability of the pooled estimate. Sequential exclusion of individual studies yielded consistently negative 95% confidence intervals, supporting the robustness of the results. No single study significantly altered the magnitude or direction of the pooled effect (Figure 8).

### 3.5 Publication bias

**Figure 9:**
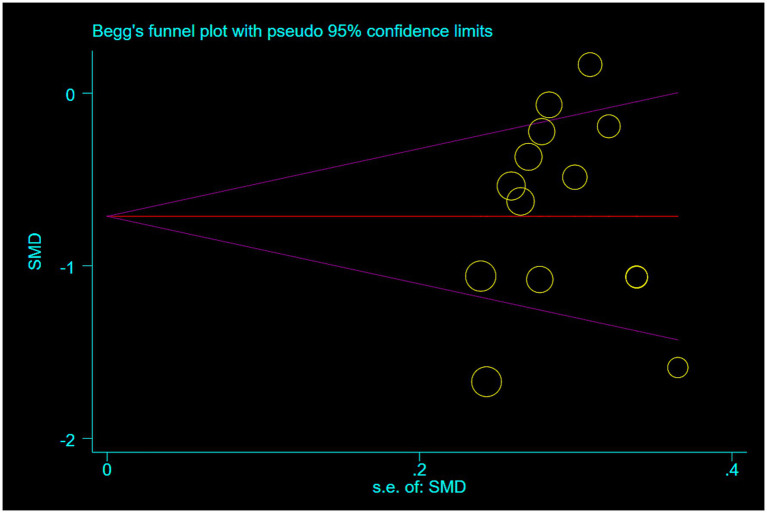
Begg’s funnel plot with pseudo 95% confidence limits.

**Figure 10:**
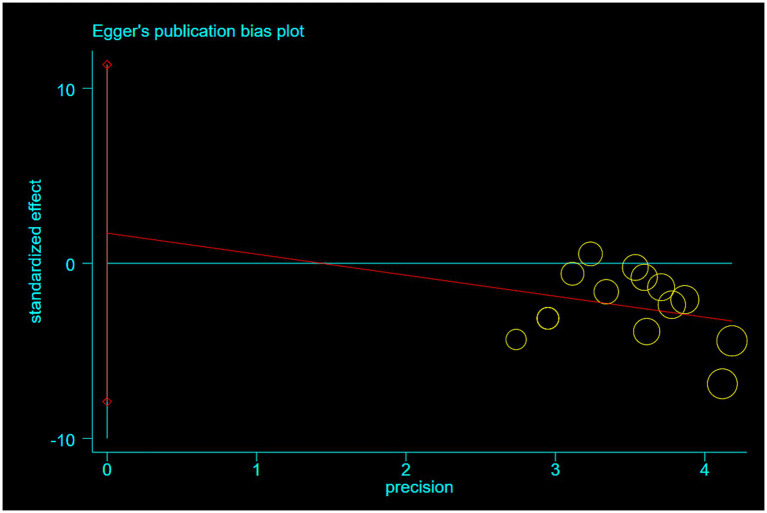
Egger’s publication bias plot.

Publication bias was assessed using Egger’s and Begg’s tests for the primary outcome of shoulder pain relief in rotator cuff injury. Both tests indicated no significant publication bias (Egger’s test: *p* = 0.703; Begg’s test: *p* = 0.584) (Figures 9, 10).

## 4 Discussion

The significant pain reduction (MD = −0.94, 95% CI: −1.27 to −0.61; *p* < 0.0001) approaches the minimal clinically important difference (MCID) for shoulder pain scales (typically 1.0–1.5 points on VAS/NRS), suggesting clinically meaningful relief (45, 46). This aligns with proposed neurophysiological mechanisms of KT, including: Gate control theory modulation: Tactile stimulation from tape may inhibit nociceptive signaling. Proprioceptive enhancement: Improved scapular positioning and neuromuscular control. Microcirculatory effects: Mechanical lift of skin facilitating lymphatic drainage and edema reduction. For functional outcomes, improvements in shoulder flexion (MD = 9.24°) and abduction (MD = 9.14°) exceed reported MCIDs for ROM in RCIs (5°–10°) (47). The greater heterogeneity in flexion (*I*^2^ = 91%) versus abduction (*I*^2^ = 38%) may reflect methodological variations in ROM assessment or biomechanical complexity of flexion kinematics. The significant differences observed, particularly in terms of pain relief and stretching efficacy, require us to interpret the aggregated results with caution and meticulousness. These differences are likely the result of the combined influence of various clinical and methodological factors. This high degree of heterogeneity indicates that the therapeutic effect of KT varies depending on specific circumstances. Although the consistent direction of the effects in the study supports its general applicability, the magnitude of the specific effects may be modulated by specific implementation methods and patient subgroups. The robust functional improvement DASH (MD = −4.38) further supports KT’s role in restoring daily activities, though its magnitude falls below the DASH MCID (10–15 points), warranting cautious interpretation (48, 49). These findings collectively present a nuanced clinical picture. The proximity of the pain reduction to the MCID threshold, coupled with the sub-MCID improvement in the DASH score, necessitates a careful distinction between statistical significance and clinical importance. The potential for publication bias was statistically assessed using Egger’s and Begg’s tests. The results indicated no significant evidence of small-study effects for the primary outcomes of pain. This meta-analysis synthesizes evidence from 17 randomized controlled trials, demonstrating that kinesiology tape (KT) significantly alleviates pain, improves shoulder mobility (flexion and abduction), and enhances upper limb function in patients with rotator cuff injuries (RCIs). The consistency of these effects across diverse outcome measures strengthens the clinical relevance of KT as a therapeutic adjunct. Yet this efficacy exists in tension with persistent methodological heterogeneity and unresolved questions regarding its mechanistic interface with the underlying pathoanatomy of rotator cuff pathology. Reconciling this duality demands a critical appraisal of KT’s action within the intricate biological and biomechanical landscape of tendon degeneration.

The subgroup analyses reveal a critical consensus: KT consistently alleviates pain across diverse clinical scenarios, with no statistically significant moderating effects observed for intervention type, treatment duration, gender distribution, or taping mechanism. The type of intervention and the duration of treatment directly influence the clinical decisions regarding how and for how long to apply KT. There was no significant gender-related modulation effect, although this needs to be interpreted with caution as it is an assessment at the research level. However, it initially suggests that the analgesic effect of KT may be generally applicable across different patient populations. This highlights its potential as a flexible intervention measure that can adapt to various clinical needs without reducing efficacy. The considerable statistical heterogeneity (*I*^2^ = 75%) prompts a more nuanced interpretation. The absence of significant subgroup effects suggests that the observed variability is likely attributable to subtler methodological and clinical distinctions rather than the predefined high-level factors. This heterogeneity may arise from a confluence of sources, such as specific technical applications of taping, the spectrum of comparator interventions (from pure placebo to active controls), and underlying clinical diversity in patient populations. Consequently, while the direction of the treatment effect is consistently positive, the high heterogeneity implies that the precise magnitude of pain relief afforded by KT is context-dependent. This underscores that the finding represents a robust trend rather than a uniform effect size and highlights the imperative for standardized reporting in future research to better delineate its optimal application. Notably, the directionality of certain trends warrants mechanistic consideration. The numerically greater pain reduction in studies with female-dominant cohorts may reflect sex-based differences in cutaneous mechanoreceptor density or central pain processing, though this requires validation via stratified RCTs. Similarly, the trend toward superior efficacy when targeting large muscle groups versus localized neuromodulation aligns with biomechanical principles: scapulothoracic stabilization fundamentally modifies rotator cuff load transmission, whereas focal sensory input primarily modulates peripheral nociception.

The pathophysiological cascade in rotator cuff injuries involves a self-perpetuating cycle: initial collagen fiber microtrauma disrupts force transmission, precipitating compensatory scapular dyskinesia and subacromial impingement. This mechanical dysfunction fuels neurogenic inflammation and nociceptor sensitization, culminating in pain-mediated movement inhibition and functional decline (50, 51). KT’s therapeutic efficacy stems from its ability to interrupt this deleterious loop through neuromodulatory and biomechanical pathways. By enhancing cutaneous proprioceptive feedback, KT refines cortical representation of scapular position, facilitating normalized recruitment of rotator cuff musculature and mitigating aberrant joint loading patterns that perpetuate tissue stress (52). For pain relief, the continuous sensory input from the tape is thought to modulate pain perception through the pain-gate theory, reducing the central transmission of nociceptive signals (53). Concurrently, its elastic recoil creates subtle subdermal convolutions, potentially augmenting lymphatic drainage and venous return within the peritendinous space, thereby dampening local inflammatory mediators (54). This mechanistic profile clarifies KT’s strength in delivering rapid symptomatic relief, similar to the transient anti-inflammatory action of corticosteroids. However, it simultaneously reveals KT’s inherent limitation: as an external biomechanical modulator, KT cannot directly resolve the core pathology of chronic rotator cuff injuries, including tendon matrix disorganization, fatty infiltration, and the intrinsic deficit in healing capacity (55, 56).

The translation of KT’s symptomatic efficacy into clinical practice thus necessitates strategic integration within a broader therapeutic paradigm. Its profound value lies not as a monotherapy aimed at structural restoration, but as a catalyst for functional engagement during the critical early phases of rehabilitation. By effectively reducing pain inhibition and improving movement confidence, KT creates a vital window of opportunity to initiate and sustain mechanoactive interventions—particularly progressive tendon-loading exercises—which are indispensable for driving collagen realignment and tensile strengthening through mechanotransduction (57, 58). This synergistic relationship explains why combined KT and exercise regimens frequently outperform either approach in isolation. Furthermore, KT’s exceptional safety profile, characterized predominantly by minor cutaneous reactions, positions it as a low-risk adjunct in contexts where corticosteroid injections pose unacceptable risks of tendon atrophy or where surgical intervention is premature or contraindicated (59). However, the persistent heterogeneity observed across trials, unmitigated even by rigorous subgroup analyses, serves as a potent reminder that KT’s application is not universally algorithmic. Its efficacy is intrinsically context-dependent, modulated by variables such as tear morphology (partial versus full-thickness), baseline kinematic deficits, and individual pain thresholds, demanding a precision-based approach rather than protocolized uniformity.

Current evidence remains limited by unresolved heterogeneity and insufficient long-term data. Furthermore, the inconsistency in the measurement results, the characteristics of the samples, and the differences in the KT application techniques may have certain impacts on this meta-analysis. To advance KT’s clinical translation, future studies must establish standardized application protocols through biomechanical modeling, while prioritizing longitudinal designs that integrate quantitative imaging biomarkers (e.g., tendon elastography, fat fraction MRI) with real-world functional outcomes. This integrated approach will ultimately clarify KT’s capacity to modify structural progression and inform precision rehabilitation strategies for rotator cuff injuries.

## 5 Conclusion

In conclusion, this meta-analysis provides evidence that KT application significantly reduces shoulder pain, improves shoulder flexion and abduction range of motion, and enhances upper limb function in patients with rotator cuff injuries in the short term. While substantial heterogeneity exists, particularly for pain and flexion outcomes, the direction of effect is consistently positive, and findings are robust to sensitivity analysis. KT represents a viable, low-risk therapeutic option. Future research should focus on standardizing protocols, understanding long-term effects, and identifying patient subgroups most likely to benefit.

## Data Availability

The original contributions presented in the study are included in the article/supplementary material, further inquiries can be directed to the corresponding author.
